# Restructured *Lactococcus lactis* strains with emergent properties constructed by a novel highly efficient screening system

**DOI:** 10.1186/s12934-019-1249-z

**Published:** 2019-11-14

**Authors:** Fulu Liu, Yating Zhang, Wanjin Qiao, Duolong Zhu, Haijin Xu, Per Erik Joakim Saris, Mingqiang Qiao

**Affiliations:** 1 Key Laboratory of Molecular Microbiology and Technology, College of Life Sciences, Ministry of Education, Nankai University, Room 301, Tianjin, China; 2 Department of Food and Environmental Sciences, University of Helsinki, Helsinki, Finland; 3 Department of Molecular Medicine, Morsani College of Medicine, University of South Florida, Tampa, FL USA

**Keywords:** *Lactococcus lactis*, Visually selectable marker, Large-scale genome deletion

## Abstract

**Background:**

After 2.83% genome reduction in *Lactococcus lactis* NZ9000, a good candidate host for proteins production was obtained in our previous work. However, the gene deletion process was time consuming and laborious. Here, we proposed a convenient gene deletion method suitable for large-scale genome reduction in *L. lactis* NZ9000.

**Results:**

Plasmid pNZ5417 containing a visually selectable marker P_*nisZ*_-*lacZ* was constructed, which allowed more efficient and convenient screening of gene deletion mutants. Using this plasmid, two large nonessential DNA regions, L-4A and L-5A, accounting for 1.25% of the chromosome were deleted stepwise in *L. lactis* 9k-3. When compared with the parent strain, the mutant *L. lactis* 9k-5A showed better growth characteristics, transformability, carbon metabolic capacity, and amino acids biosynthesis.

**Conclusions:**

Thus, this study provides a convenient and efficient system for large-scale genome deletion in *L. lactis* through application of visually selectable marker, which could be helpful for rapid genome streamlining and generation of restructured *L. lactis* strains that can be used as cell factories.

## Introduction

With a crucial role in dairy and health industries, *Lactococcus lactis* is a GRAS (generally regarded as safe) microorganism [1]. Features such as lack of immunogenic lipopolysaccharides and secretion of only one major protein [2] make *L. lactis* the commonly used microorganism in traditional food fermentation. Nowadays, with the development of whole genome sequencing and functional genomics technology, abundant data, including whole genome sequence and metabolic pathway of *L. lactis*, are available, which allow using *L. lactis* as an “efficient cell factory” for recombinant protein production and secretion [2]. However, an efficient genetic engineering system for *L. lactis* is still missing.

In recent decades, synthetic biology approaches have been used to improve the growth rate and other characteristics of bacteria, including *Escherichia coli* [3–7], *Pseudomonas putida* [8, 9], and *Bacillus subtilis* [10, 11], for industrial applications [12, 13]. In our previous study, 2.83% genome reduction was accomplished in *L. lactis* NZ9000 [14]. Various genome engineering tools have been constructed and used for genome editing in *L. lactis*, such as site-specific integration based on homologous recombination [15], marker-free method for chromosomal mutations/deletions using ssDNA oligo’s Cre/*loxp* recombination system [16, 17], and CRISPR–Cas9/CRISPR-based genome editing system [17, 18]. The most recently reported tool that allows multiple genes and large-scale genome deletion in *L. lactis* is two-plasmid (pNZ5319 and pNZTS-Cre) and Cre/*loxp* based site-specific recombination system [14, 19]. Plasmid pNZ5319 [20] was used to construct knock-out vectors, which was subsequently transformed into *L. lactis* and replaced the target gene with cassette *lox66*-P_32_-*cat*-*lox71* through double-crossover recombination. Then, temperature-sensitive plasmid pNZTS-Cre [19] for Cre recombinase expression was transformed into the mutants, which integrated with the *lox66*-P_32_-*cat*-*lox71* cassette. The Cre recombinase excised the *cat* gene through the two *lox* sites (*lox66* and *lox71*) and generated a double-mutant *loxP* site (*lox72*), which displayed strongly reduced recognition by Cre recombinase [20], finally, pNZTS-Cre was cured through the shift of temperature [19]. However, screening of the second-crossover recombination through replica plating method is laborious and time-consuming.

In the present study, a convenient gene deletion system was established by replacing plasmid pNZ5319 with a new plasmid containing a selectable marker P_*nisZ*_-*lacZ.* This enabled identification of deletions through visual screening, which facilitated quicker and easier detection of deletion of genes in *L. lactis*. With this system, two large nonessential DNA regions (L-4A and L-5A) accounting for 1.25% of the genome were selected and deleted stepwise in *L. lactis* 9K-3 [14] with high efficiency, and ultimately, five large nonessential DNA regions deletion mutant *L. lactis* 9K-5A with 3.24% genome reduction was constructed. To explore the genetic potential of mutants, the whole genome of *L. lactis* 9K-5A was sequenced. Comparison of physiological traits and transcriptome analysis revealed that *L. lactis* 9k-5A outperformed the wild strain in several physiological traits assessed and exhibited much higher expression of genes involved in routine metabolism.

## Results

### Construction of vector pNZ5417

**Fig. 1:**
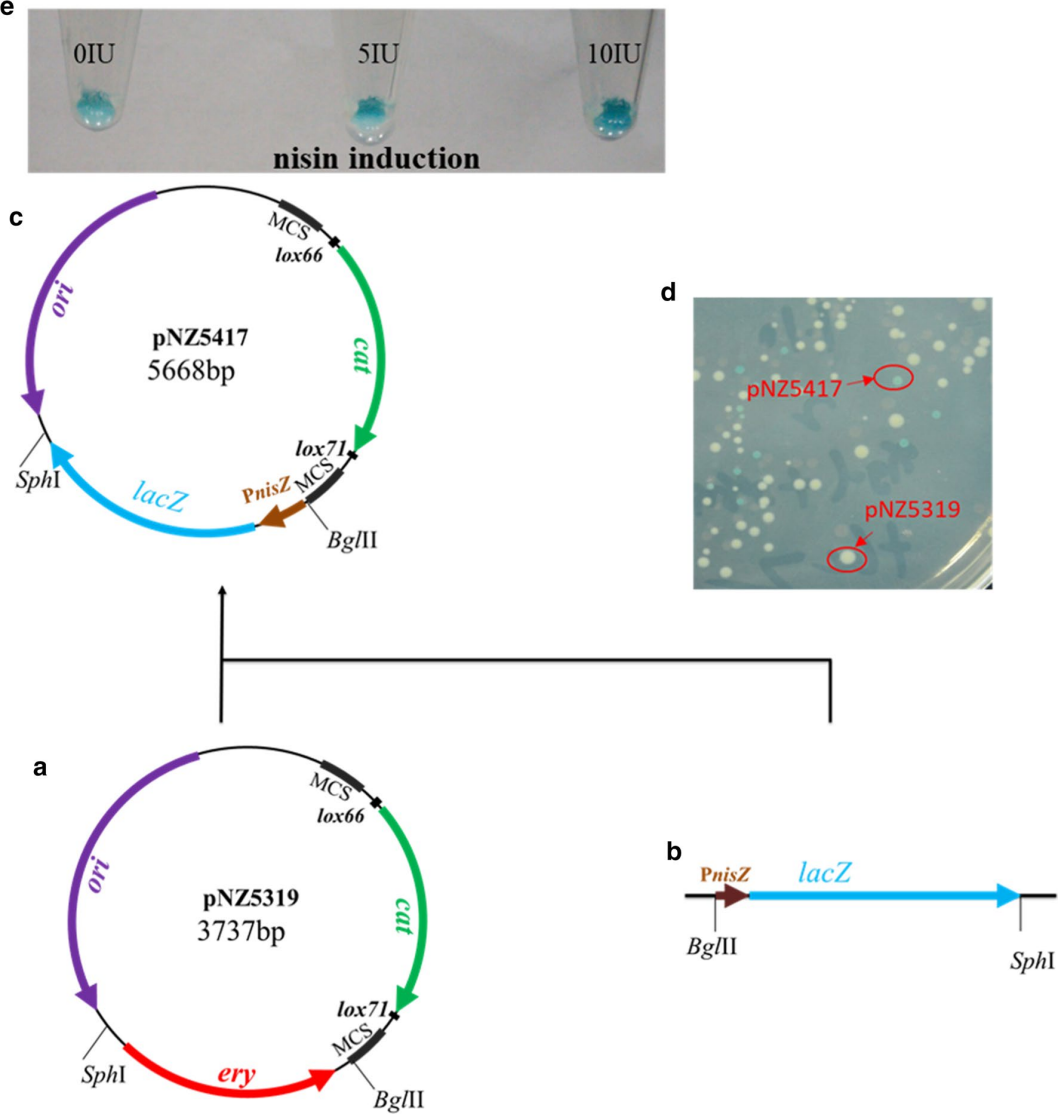
Schematic representation of deletion plasmid pNZ5417 construction. **a** Scheme of pNZ5319; **b** color selectable marker gene cassettes (P_*nisZ*_-*lacZ*); **c** scheme of pNZ5417; **d** chromogenic reaction of pNZ5417 in *E. coli* on LB medium containing chloramphenicol and X-gal; **e** chromogenic reaction of *L. lactis* NZ9000 harboring pNZ5417Δ L4A in M17 medium containing chloramphenicol, X-gal, and gradient nisin

Plasmid pNZ5417, based on pNZ5319, was constructed by replacing *ery* with *lacZ* gene under the control of nisin-inducible promoter P_*nisZ*_, which produced blue colony on LB plate containing chloramphenicol and X-gal LB-CX (Fig. 1d).

### Evaluation of the new gene deletion system in *L. lactis* NZ9000

**Fig. 2:**
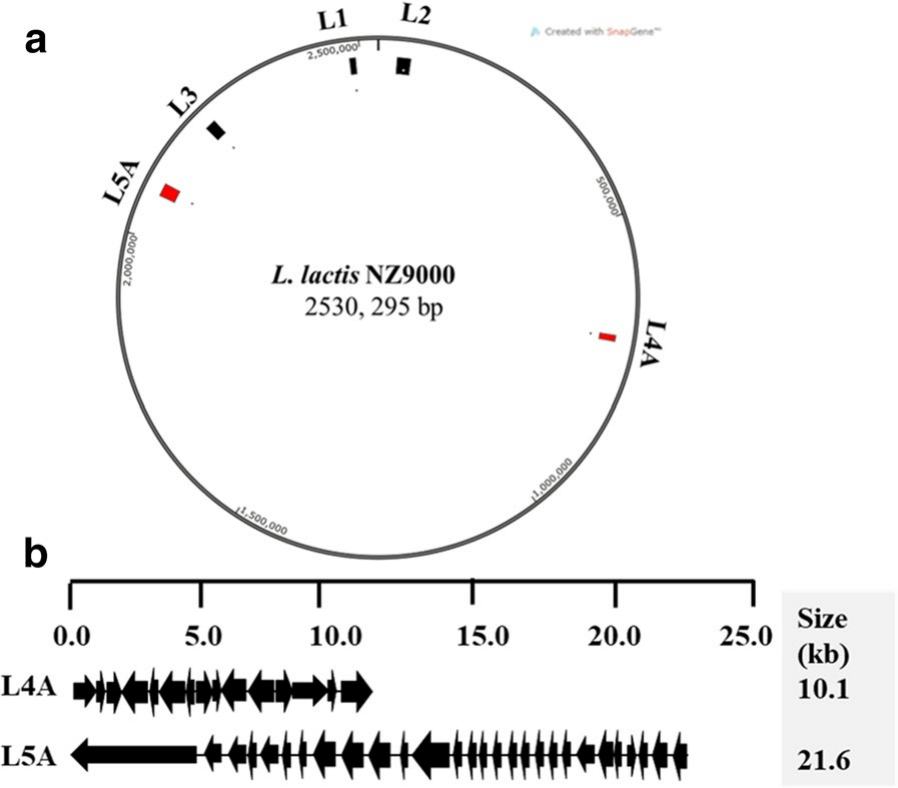
Genetic organization and deletion of two large nonessential DNA regions in *L. lactis* 9k. **a** Circular map of *L. lactis* 9k chromosome showing the physical location of deletions in the genome; **b** genetic organization of two large deletions

To evaluate the feasibility of pNZ5417 for large-scale gene deletion in *Lactococcus* strains, two large nonessential DNA regions, L4A and L5A, were successfully deleted stepwise in *L. lactis* 9k-3. The distribution of L4A and L5A throughout the genome is indicated in Fig. 2a. The genetic organization of the two deleted DNA regions is shown in Fig. 2b. A detailed description of genes included in the L4A and L5A regions is provided in Additional file 1: Table S1. These two DNA regions formed approximately 1.25% of the *L. lactis* 9k genome, as shown in Table 1.

**Table 1:** Summary of deletion strains based on updated NZ9000 sequence (CP002094.1, Linares DM 2010)

Strains	Deletion units	Removed (bp)	Cumulative (bp)	Deletion (%)	Doubling time (min)
9K	0	0	0	0	46.6
*L. lactis* 9K-1	17	9740	9740	0.374	NA
*L. lactis* 9K-2	40	22,518	32,258	1.26	NA
*L. lactis* 9K-3	34	17,905	50,163	1.986	NA
*L. lactis* 9K-4A	15	10,090	60,253	2.385	43.2
*L. lactis* 9K-5A	31	21,624	81,877	3.235	43.3

**Fig. 3:**
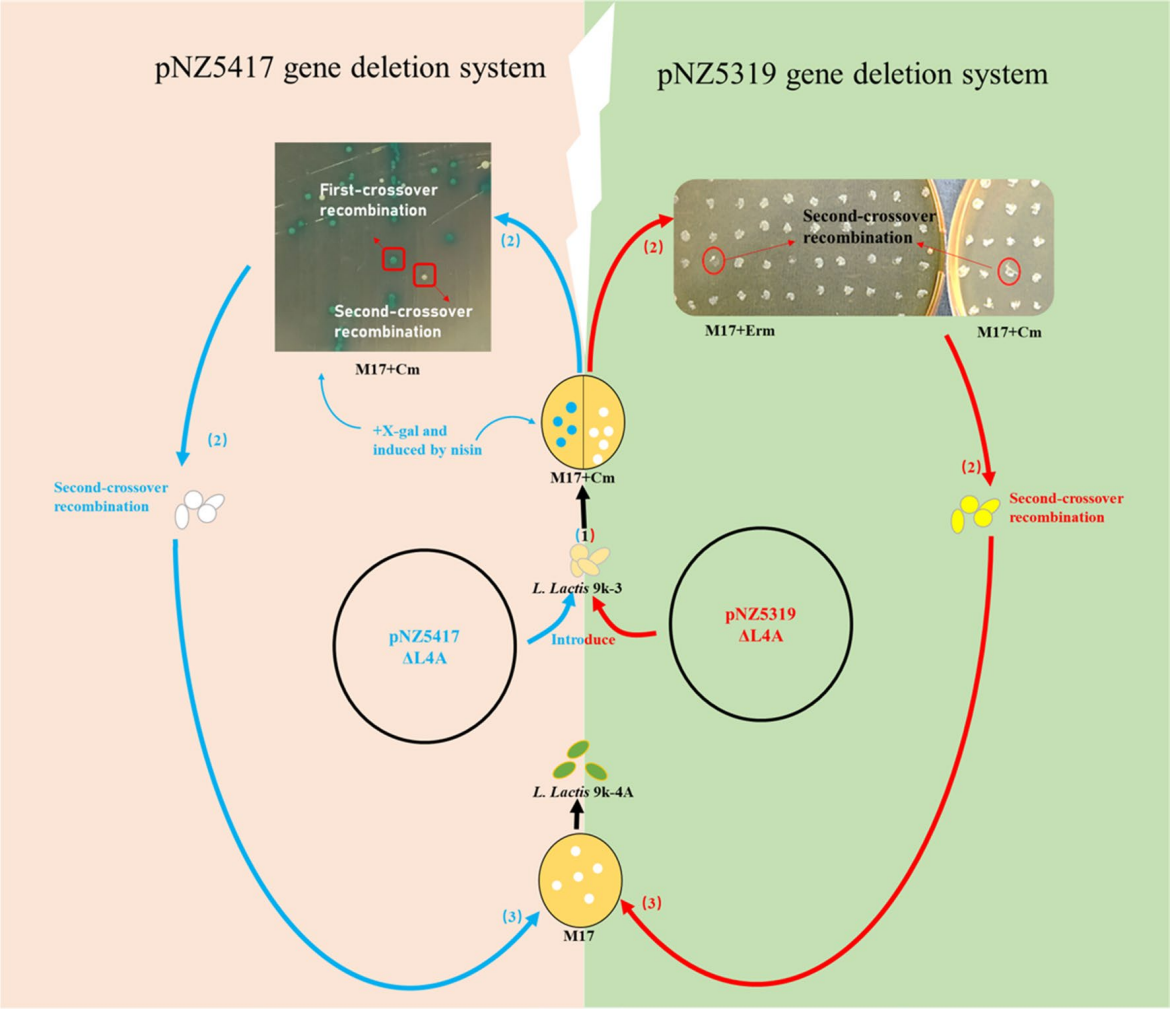
Overall scheme for L4A deletion using two systems in *L. lactis* 9k. (1) Plasmid pNZ5319ΔL4A or pNZ5417ΔL4A was first loaded into *L. lactis* 9k-3 cells and the recombinants were selected on M17 plates supplemented with 5 μg/mL chloramphenicol (Cm); for pNZ5417ΔL4A, 40 μg/mL X-gal and 10 IU/mL nisin were also added; (2) recombinants harboring plasmid pNZ5319ΔL4A or pNZ5417ΔL4A were cultured in M17 medium supplemented with 5 μg/mL chloramphenicol for generations, and positive mutants with successful L4A deletion were selected by replica plating method (for “pNZ5319/pNZTS-Cre gene deletion system”) or color change (for “new gene deletion system”); (3) deletion of the chloromycetin resistance marker and elimination of temperature-sensitive plasmid pNZTS-Cre [19]

To prove the higher deletion efficiency of plasmid pNZ5417, L4A was also deleted with plasmid pNZ5319 (Fig. 3). The deletion mutants were generated with both the deletion plasmids through double-crossover recombination and the following three steps were executed to obtain genes deletion mutants: (i) the deletion vector was constructed and the first-crossover recombination was accomplished; (ii) the cells were cultured and second-crossover recombination was achieved; and (iii) chloramphenicol resistance marker was deleted, and after it, plasmid pNZTS-Cre was cured. When compared with pNZ5319, 65% of the time was saved at the screening of second-crossover recombination, pNZ5417 gene deletion system allowed mutants screening based on color change, which is convenient and time-saving compared to the replica plating method.

### Assessment of growth profiles and transformability of mutants

**Fig. 4:**
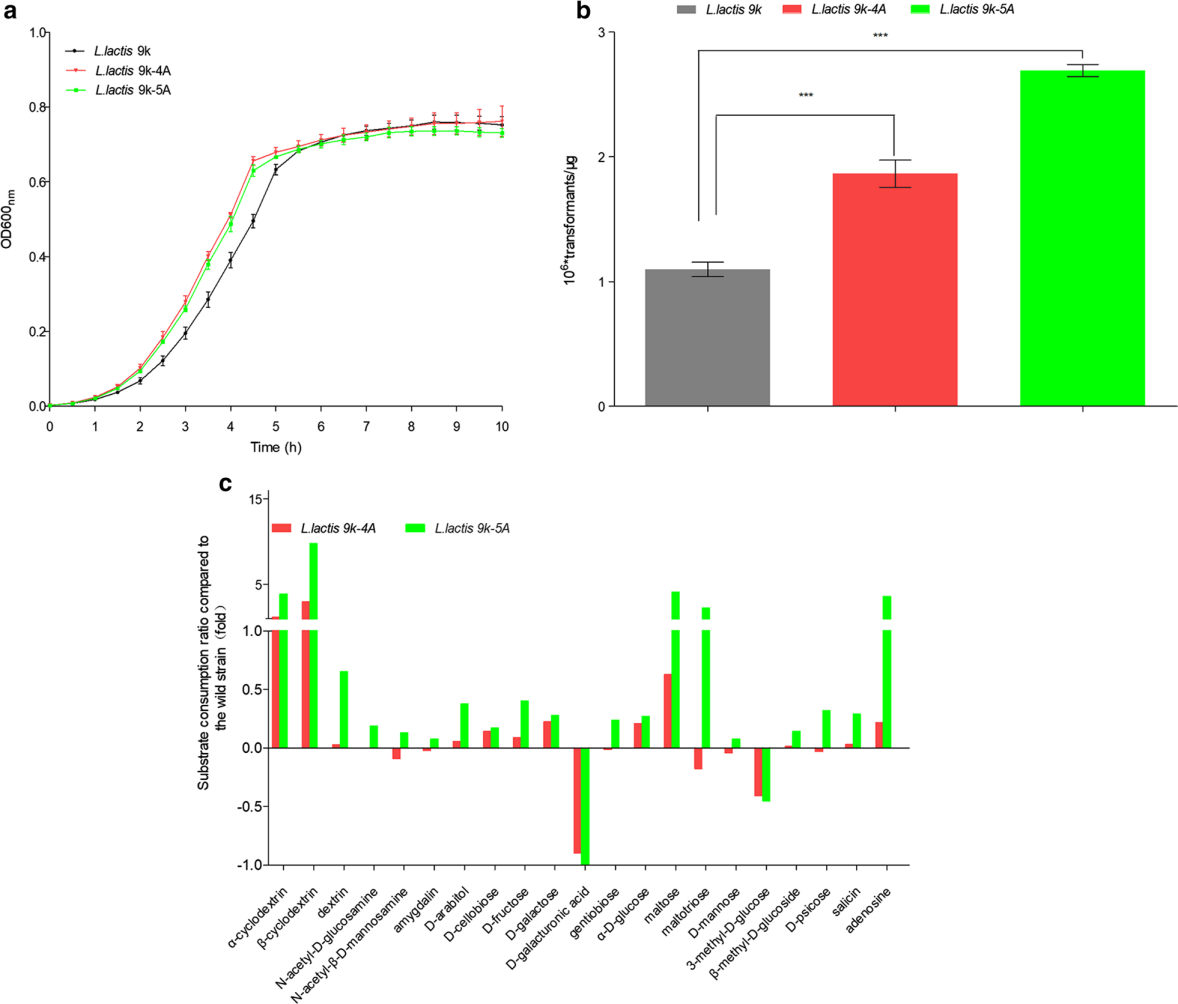
Characterization of lactococcal strains. **a** Growth profile analysis with M17 medium; **b** comparison of electroporation efficiency. Data showed as mean ± SD and compared by *t*-test, ***P < 0.001. **c** Assessment of extensive fermentation phenotype of the mutants

The growth profiles of *Lactococcus* strains (*L. lactis* 9k, *L. lactis* 9k-4A, *L. lactis* 9k-5A) were monitored by recording the OD_600_ in GM17, respectively. The growth curves are shown in Fig. 4a (GM17). The results revealed that both the mutants grew much faster in the exponential phase, and reached the lag growth phase 1 h earlier than the parent strain. The doubling time of the two mutants proved to be much shorter than that of *L. lactis* 9k in the exponential growth phase (Table 1). As reported before, genome evolution should promote an enlarged genome size by incorporating non-essential accessory genes [21, 22], which might be disadvantageous for the growth fitness, given the additional cost for the replication and expression of the newly acquired sequences. After genome reduction, accumulative loss of dispensable genomic sequences like pseudogenes, phage/IS and unknown function genes contributes to bacterial growth in a dose-dependent manner [23]. Thus, we suppose the losing of unknown nonessential genes cased faster growth of mutants.

The electroporation efficiency of all the strains was measured by electroporating a small supercoiled plasmid, pNZ8048, into the cells. As shown in Fig. 4b, both mutants *L. lactis* 9k-4A (1.70-fold, *P *< 0.001) and *L. lactis* 9k-5A (2.45-fold, *P *< 0.001) exhibited higher electroporation efficiency than the parent strain (1.10 × 10^6^/μg plasmid DNA). GyöRgy et al. [7] reported that removal of external structures and unknown deoxyribonuclease or restriction system or activation of an unknown DNA uptake factor could affect the recovery of transformants. Considering the results of transcriptome analysis (Additional file 2: Fig. S1), we suppose deletion of unknown genes affected the cellular component including membrane composition, and altered the ability of mutants to receive exogenous DNA.

### Assessment of mutants’ phenotype

Extensive fermentation phenotype analyses of *L. lactis* 9k, *L. lactis* 9k-4A, and *L. lactis* 9k-5A were conducted using the phenotype microarrays to explore the physiological difference between the wild and mutant *Lactococcus* strains. All of the substrates that the mutants consumed were significantly different from those of *L. lactis* 9k. The result shown in Fig. 4c revealed that *L. lactis* 9k-4A can efficiently metabolize 12 carbon sources, particularly, α-cyclodextrin, β-cyclodextrin, and maltose; while *L. lactis* 9k-5A effectively metabolized 19 carbon sources, among which metabolism of α-cyclodextrin, β-cyclodextrin, maltose, maltotriose, and adenosine was 4.9-, 10.8-, 5.1-, 3.2-, and 4.6-fold higher than that of wild strain, respectively. We suppose this make it possible for mutants to utilize more carbon sources as the sole carbon source, especially α-cyclodextrin and β-cyclodextrin, as a member of oligosaccharide, they are much easier to get and cheaper carbon source than glucose. In contrast, both the mutants showed poor capacity to metabolize d-galacturonic acid and 3-methyl-d-glucose, with *L. lactis* 9k-5A losing its ability to metabolize d-galacturonic acid.

### Genome sequencing and analysis of *L. lactis* 9k-5A

The genome of mutant *L. lactis* 9k-5A was sequenced by Shanghai Majorbio Bio-pharm Technology Co. (Shanghai, China) using the Illumina MiSeq platform. As shown in Additional file 3: Data S1A, the final assemble consisted of 91 scaffolds with a total size of 23,33,697 bp and 35.61% G+C content, including 66 large scaffolds with the largest scaffold comprising 396,482 bp. Furthermore, 2390 genes with a total length of 1,985,466 bp were predicted and annotated. The putative replication origins of *L. lactis* 9k-5A were localized by GC skew (Additional file 4: Data S2A). The 66 large scaffolds of *L. lactis* 9k-5A were arranged (Additional file 5: Data S1B) in the order of genome sequence of *L. lactis* NZ9000, and then subjected to BLAST (National Center for Biotechnology Information; https://blast.ncbi.nlm.nih.gov/Blast.cgi?PROGRAM=blastn&PAGE_TYPE=BlastSearch&LINK_LOC=MultiSensor). Sequence alignment indicated that the five nonessential DNA regions L1, L2, L3, L4A, and L5A were successfully deleted (Additional file 6: Data S2B).

### Transcriptome assessment of mutants

Gene expression of *L. lactis* 9k, *L. lactis* 9k-4A, and *L. lactis* 9k-5A was analyzed using transcriptome analysis, and differentially expressed genes (DEGs; false discovery rate ≤ 0.001 and |log2| ≥ 1) were identified and subjected to Gene Ontology enrichment analysis (Additional file 7: Fig. S2A–C). When compared with *L. lactis* 9k, 117 genes were upregulated and 75 genes were downregulated in *L. lactis* 9k-4A (Additional file 7: Fig. S2D), and the expression of genes located in the four nonessential DNA regions L1, L2, L3, and L4A was significantly downregulated (Additional file 8: Table S2). In *L. lactis* 9k-5A, 245 genes were upregulated and 93 genes were downregulated (Additional file 7: Fig. S2D), and the expression of genes in the five nonessential DNA regions L1, L2, L3, L4A, and L5A was significantly downregulated, when compared with those in *L. lactis* 9k (Additional file 8: Table S2).

**Fig. 5:**
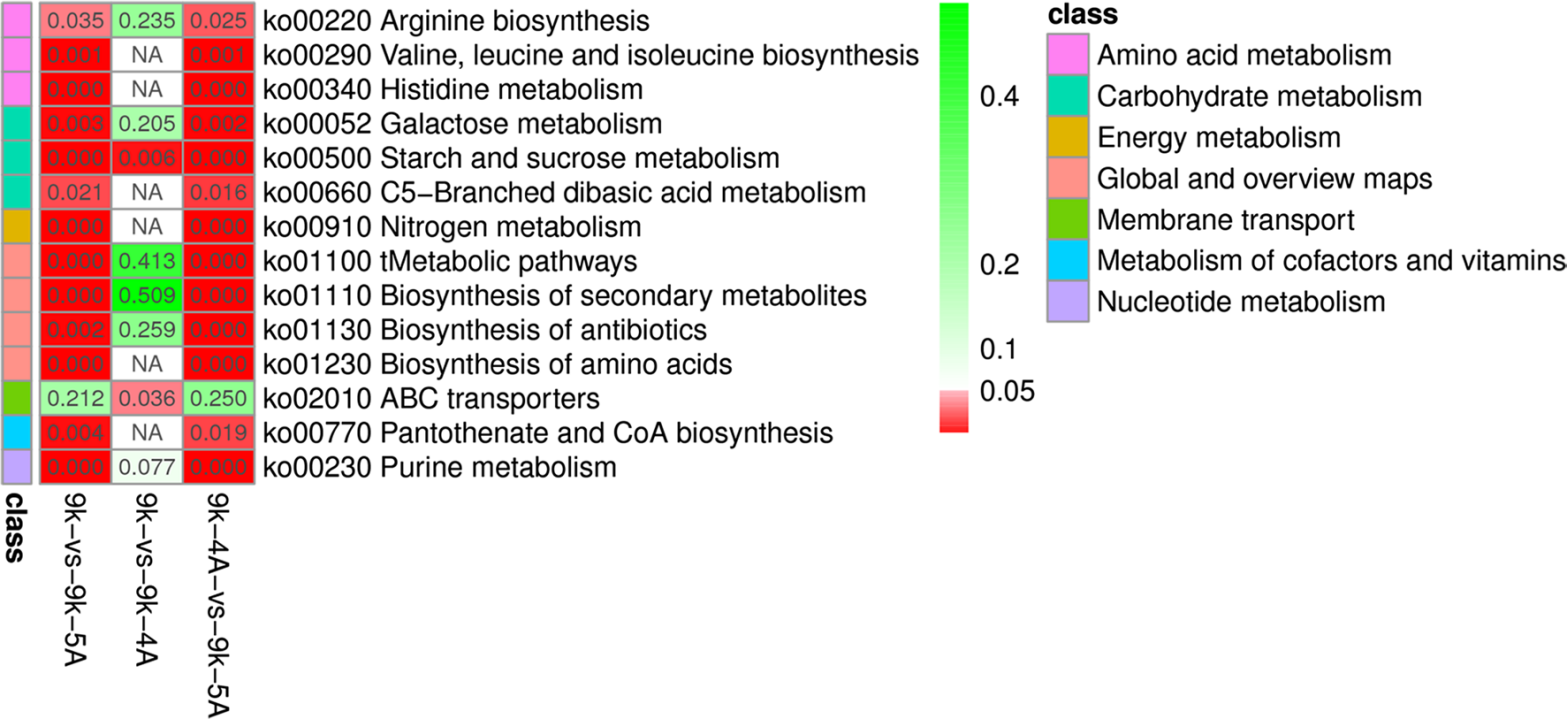
Significant enrichment of KEGG pathway for selected DEGs in *L. lactis* 9K-4A and *L. lactis* 9K-5A. KEGG pathway with *P* < 0.05 is highlighted, indicating significant enrichment in DEGs

With the exception of genes with low expression level or located in the deleted nonessential DNA regions, 93 DEGs (FDR < 0.05) in the mutants were selected and analyzed in this study (Additional file 9: Fig. S3 and Additional file 10: Table S3). When compared with *L. lactis* 9k, biosynthesis of amino acids, purine metabolism, starch and sucrose metabolism, and some other pathways were significantly enriched in *L. lactis* 9k-5A (Fig. 5). As shown in Additional file 11: Data S3A, *malL* and *malP* genes involved in the metabolism of maltose, and *malD*, *mdxF*, and *malX* genes that participate in the transformation of maltotriose were significantly upregulated in *L. lactis* 9k-5A, which were in agreement with the GP2 MicroPlate results (Fig. 4c). Besides, the expression levels of genes involved in the synthesis of valine and isoleucine (*ilvA*, *ilvB, ilvC*, *ilvD*, *ilvH*), histidine (*hisA*, *hisB*, *hisC*, *hisD*, *hisF*, *hisG*, *hisH*, *hisI*, *hisK*, *hisZ*), and genes involved in glutamine metabolism (*gltA*, *gltB*, *purC*, *purD*, *purE*, *purF*, *purK*, *purL*, *purM*, *purN*, *purQ*, *purS*) were also upregulated in *L. lactis* 9k-5A (Additional file 11: Data S3A, Additional file 12: Data S3B and Additional file 13: Data S3C).

Subsequently, four genes with higher expression levels were examined by RT-qPCR to check the transcriptome data. When compared with *L. lactis* 9k, the expression levels of genes *malD*, *purF*, *galE*, and *galK* were only slightly changed in *L. lactis* 9k-4A (about 2.2-, 0.8-, 0.5-, and 0.5-fold, respectively), but were upregulated in *L. lactis* 9k-5A (up to 27.2-, 12.7-, 3.5-, and 7.9-fold, respectively) (Additional file 14: Fig. S4), which were in agreement with the results of transcriptome assay.

## Discussion

With the rapid development of metabolic engineering, genome editing in bacteria, including industrial microorganisms such as *E. coli* [4, 24] and *B. subtilis* [25], has provided significant benefits. Xin et al. [26, 27] reported a single-plasmid genome editing system in lactic acid bacteria, which offered convenient and easy-to-use genome-editing tool for metabolic engineering in *Lactobacillus casei*. Guo et al. [17] established a rapid tool for genomic engineering by combining ssDNA recombinants with improved CRISPR/Cas9 counter selection, and achieved seamless genomic DNA deletions (50/100 bp) in *L. lactis*. However, sequential deletion of multiple genes and large-scale genome in *L. lactis* is still a time-consuming and laborious process. In the present study, we proposed a convenient system for sequential generation of combinatorial genome deletions in *L. lactis*.

Counter selection method based on homologous recombination is a convenient and efficient technique for *L. lactis* genome streaming [28, 29]. Nevertheless, the percentage of revertant mutations (60–92%) is much higher in double-crossover mutants [28], which makes the whole process of gene deletion much laborious. In our previous study, a two-plasmid (pNZ5319 and pNZTS-Cre) based gene deletion system was established with 100% correct deletion efficiency [14, 19]. However, laborious procedures were still needed to screen the second-crossover recombination through replica plating method (Fig. 3, Additional file 15: Table S4).

In the present study, the screening time decreased by 38% with the developed gene deletion system (Additional file 15: Table S4). Our proposed system comprised a visually selectable marker *lacZ*, which was under the control of inducible promoter P_*nisZ*_ [30]. While the chromogenic reaction became more significant with the induction of nisin, the presence of original constitutive promoter P_32_ in “NZ9000 and MG1363 harboring pNZ5417ΔL4A” turned the cells blue in GM17 medium containing chloramphenicol and X-gal without nisin induction (Fig. 1e, Additional file 16: Fig. S5). Therefore, the use of this plasmid is not limited to nisin-controlled gene expression (NICE) system.

Two large nonessential DNA regions (L4A and L5A) of the *L. lactis* NZ9000 genome were deleted sequentially with our developed gene deletion system, and subsequently, a five large nonessential DNA regions (3.24% of the genome) deletion mutant was constructed. When compared with the parent strain, the two mutants, *L. lactis* 9k-4A and *L. lactis* 9K-5A, showed some good phenotypic changes, including better growth characteristics and transformability. The capability of the strains in metabolizing 95 carbon sources was compared using GP2 MicroPlate, which revealed that *L. lactis* 9k-4A and *L. lactis* 9k-5A had better capacity to metabolize 12 and 19 carbon sources, respectively. The results of transcriptome analysis indicated that 245 genes were upregulated and 93 genes were downregulated in *L. lactis* 9k-5A. The selected 93 DEGs showed significant enrichment of KEGG pathway, which indicated a much higher expression of *malL*, *malP*, *malD*, *mdxF*, and *malX* genes involved in the metabolism of maltose and transformation of maltotriose, suggesting that *L. lactis* 9k-5A had better ability to metabolize maltose and maltotriose, similar to the results of GP2 MicroPlate. Besides, genes involved in the pathway of histidine, valine, and isoleucine biosynthesis and some other pathways were significantly upregulated in *L. lactis* 9k-5A, implying that this mutant could be employed as a possible industrial cell factory for the production of these three amino acids.

## Conclusion

To the best of our knowledge, this study is the first to introduce inducible visually selectable marker P_*nisZ*_-*lacZ* into *L. lactis* NZ9000 gene deletion system with improved efficiencies of 38% in achieving gene deletion mutants, which will save much more time in genome reduction. By using this system, two nonessential DNA regions were deleted sequentially in *L. lactis*. Our main contributions, in addition to the improved gene deletion system, was the final genome-streamlined mutant *L. lactis* 9k-5A exhibited good phenotypic changes, including better growth characteristics, transformability, carbon metabolic capacity, and biosynthesis of amino acids. The results of this study indicated that further genome refinements and reductions in *L. lactis* could eventually generate a significantly simplified strain that could contribute to broadening the use of this bacterium.

## Methods

### Bacterial strains, plasmids, and culture conditions

The strains and plasmids used in this study are listed in Table 2. *L. lactis* was grown at 30 °C under static condition in GM17 medium supplemented with 0.5% (w/v) glucose. *E. coli* DH5α cells were used as cloning host and grown aerobically at 37 °C in LB medium (1% tryptone, 0.5% yeast extract, and 1% NaCl; the solid medium contained 1.5% agar). Antibiotic selection was used when appropriate: for *E. coli* (per mL), 150 μg of erythromycin and 15 μg of chloramphenicol were employed and for *L. lactis* (per mL), 5 μg of erythromycin and 5 μg of chloramphenicol were applied. X-gal was used at a concentration of 80 μg/mL and nisin was utilized at a concentration of 10 IU/mL.

**Table 2:** Bacterial strains and plasmids utilized in this study

Item	Genotype of phenotype	References
Strains		
*E. coli* DH5α	Cloning host; F -φ80*lacZ*∆M15*endA1 recA1 endA1 hsdR17* (rK-mK +)*supE44 thi*-*1 gyrA 96 relA1* ∆ *(lacZYA*^−^*argF)U169 deoR λ*^−^	[39]
*L. lactis* N8	Nisin Z producing strain	[40]
*L. lactis* MG1363	*L. lactis* subsp. *cremoris*, plasmid-free derivative of NCDO712	[41]
*L. lactis* NZ9000 (9k)	MG1363 *pepN::nisRK*, the original strain	[42]
*L. lactis* 9k-3	Three fragments (about 50 kb) deletion in *L. lactis* 9K	[14]
*L. lactis* 9k-4A	The L4A (about 10 kb) deletion in *L. lactis* 9k-3	This work
*L. lactis* 9k-5A	The L5A (about 21.5 kb) deletion in *L. lactis* 9k-4A	This work
Plasmids		
pNZ5319	Cm^r^, Em^r^, the original vector	[20]
pNZ5319Δ L4A	Cm^r^, Em^r^, L4-A knock-out vector	This work
pNZ5417	Cm^r^, Derivative of pNZ5319; Δ*ery*; containing *P*_*nisZ*_*::lacZ*	This work
pNZ5417Δ L4A	Cm^r^, L4-A knock-out vector	This work
pNZ5417Δ L5A	Cm^r^, L5-A knock-out vector	This work
pNZTS-Cre	Em^r^, *cre* gene cloned at the *EcoR*I and *Hind*III sites	[19]
pNZ8048	Cm^r^	[42]

### DNA manipulations and chemicals

DNA marker, T4 DNA ligase, restriction enzymes, and DNA gel extraction kit were purchased from Takara (Dalian, China). The PCR product purification kit, first-strand cDNA synthesis kit, and SYBR Green RT-qPCR kit were obtained from Thermo Fisher Scientific (Waltham, USA). The commercial X-gal and nisin were bought from Sigma-Aldrich (St. Louis, USA). *L. lactis* plasmid DNA, chromosomal DNA, and total RNA were isolated by using Qiaprep spin kit (small scale) following manufacturer’s instructions. PCR was performed with Phusion enzyme (Thermo Fisher Scientific, Waltham, USA). Primers were synthesized by BGI (Beijing, China) and the corresponding sequences are listed in Additional file 17: Table S5. PCR products and plasmids were sequenced by GENEWIZ service (Hangzhou, China). The competent *E. coli* DH5α cells were purchased from Takara (Dalian, China) and transformed by CaCl_2_ procedure [31]. Recombinant plasmids were introduced into *L. lactis* by electroporation according to the method described earlier [32].

### Construction of vector pNZ5417

The plasmid pNZ5417 (Fig. 1c), containing *lacZ* gene under the control of nisin-inducible promoter P_*nisZ*_ [30], was constructed from pNZ5319. Promoter P_*nisZ*_ and *lacZ* gene were obtained from *L. lactis* N8 with primer pairs P_*nisZ*_-F/R and LacZ-F/R, combined by overlap PCR (Fig. 1b), and digested with *Bgl*I–*Sph*I, and replaced the *ery* gene of pNZ5319 to generate pNZ5417.

### Feasibility of new gene deletion system in *L. lactis* NZ9000

To evaluate the new gene deletion system, we deleted the large nonessential DNA region L4A in *L. lactis* 9k-3 [14] by using pNZ5319/pNZTS-Cre [19] and pNZ5417/pNZTS-Cre gene deletion system, respectively, and successfully constructed *L. lactis* 9k-4A. Gene knock-out vectors pNZ5319Δ L4A and pNZ5417Δ L4A were generated with the primer pairs L4A-UP-F/R and L4A DP-F/R. Vector pNZ5319Δ L4A was transformed into *L. lactis* 9k-3, and the deletion of L4A mutant with pNZ5319/pNZTS-Cre system was achieved as described earlier [19]. pNZ5417Δ L4A was transformed into *L. lactis* 9k-3, and single cross-over recombinant was selected at 30 °C on GM17-CXN solid medium containing chloramphenicol, X-gal, and nisin. The single cross-over recombinants were sub-cultured at 30 °C in GM17-CXN liquid medium several times, and the overnight cultures were diluted and plated on GM17-CXN medium at 37 °C until most colonies turned blue. The white colonies were selected and identified by primer pairs L4A Int-F/R and L4A Out-F/R. After single colony isolation, the *cat* selectable marker was excised as described previously, and the deletion mutants were tested by PCR with appropriate primers. The gene knock-out vector pNZ5417Δ L5A was constructed with primer pairs L5A-UP-F/R and L5A-DP-F/R, and the nonessential DNA region L5A was deleted in *L. lactis* 9k-4A with pNZ5417 gene deletion system to obtain five large nonessential DNA regions deletion mutant *L. lactis* 9k-5A.

### Analysis of growth profiles

*Lactococcus lactis* 9k, *L. lactis* 9k-4A, and *L. lactis* 9k-5A were cultured to OD_600_ of 0.8 in GM17 medium and diluted to OD_600_ of 0.4. Then, 2 µL of the diluted cultures were reinoculated into 200 µL of GM17 medium in shake-flasks. The growth profiles were monitored by measuring OD_600_ for 10 h at 30 °C by using a Bioscreen machine (Lab-systems, Helsinki, Finland) [33]. The experiment was repeated three times.

### Measurement of electroporation efficiency

Electrocompetent cells of all the strains were prepared by the method of Holo [32], and 2.5 µg of plasmid pNZ8048 DNA were added to 0.1 mL of competent cells. After electroporation, the cells were cultured in plates containing 15 µg/mL chloramphenicol for the selection of chloramphenicol-resistant transformants. The transformants were enumerated after 2 days of incubation at 30 °C, and the experiment was repeated three times.

### Microarray analysis of mutants’ phenotype

The metabolism of the wild strain and mutants was examined with GP2 MicroPlate™ using phenotype microarrays system (Biolog, California, USA). Sample preparation and assays were conducted according to the manufacturer’s instructions. In brief, *Lactococcus* cells on the surface of solid medium were collected using cotton swab and suspended in inoculating fluid (0.40% NaCl, 0.03% Pluronics F-68, and 0.02% Gellan Gum) (Biolog, California, USA). The cell density was equalized, and 150 µL of the cells suspension were pipetted into GP2 plates with various substrates, respectively. Then, the plates were incubated in OmniLog^®^ instrument (Biolog, California, USA) at 30 °C for 24 h. The data were automatically recorded every 30 min, and were analyzed by OL-OM software (version 3.0) (Biolog, California, USA).

### Sequencing and analysis of *L. lactis* 9k-5A genome

The genomic DNA of *L. lactis* 9k-5A was extracted and purified, and then quantified using Nanodrop 2000 spectrophotometer (Thermo Scientific, USA). The *L. lactis* 9k-5A genome was sequenced by Shanghai Majorbio Bio-pharm Technology Co. (Shanghai, China) using Illumina MiSeq platform with a paired-end library. Following trimming and merging, the reads were assembled de novo using SOAP denovo V2.04 [34]. Open reading frames (ORFs) were predicted using Glimmer 3.02 program [35], and annotated by comparison with NCBI-NR and KEGG databases using BLASTp (BLAST 2/2/28+). Furthermore, tRNA and rRNA were predicted using tRNA scan-SE v. 1.3.1 and Barr nap 0.4.2 (www.vicbioinformatics.com/software.barrnap.shtml) programs, respectively [36].

### Transcriptome analyses of mutants

The total RNA of *L. lactis* 9k, *L. lactis* 9k-4A, and *L. lactis* 9k-5A strains cultured in GM17 to an OD_600_ of 0.8 was extracted and purified by TRIzol kit (Promega USA), sequenced on Illumina sequencing platform, and analyzed by Genedenovo Biotechnology Co., Ltd (Guangzhou, China). Each sample was prepared in triplicate. The transcription of genes *malD*, *purF*, *galE*, and *galK* was measured through quantitative real-time PCR (RT-qPCR) to recheck the transcriptomic data. All RT-qPCR reactions were repeated independently three times. Data analysis was conducted by using comparative CT (2^−∆∆CT^) method with the housekeeping gene *rpoB* [37] as control. Transcription with more than twofold changes was regarded as significant difference [38].

### Statistical analysis

The data obtained are reported as mean ± standard deviation (SD). The difference between two groups was compared by *t*-test with *P* < 0.05 considered as significant.

## Supplementary information

**Additional file 1: Table S1.** Gene content of deleted DNA regions.

**Additional file 2: Figure S1.** Go terms analysis for all the DEGs of all mutants. (A) Go terms of *L. lactis* NZ9000-VS-*L. lactis* 9k-4A, (B) *L. lactis* NZ9000-VS-*L. lactis* 9k-5A, (C) *L. lactis* 9k-4A-VS-*L. lactis* 9k-5A.

**Additional file 3: Data S1A.** Genome sequencing and analysis of *L. lactis* 9k-5A. (A) Genome sequencing of *L. lactis* 9k-5A.

**Additional file 4: Data S2A.** Circular graph of *L. lactis* 9K-5A (A) and alignment with parent strain *L. lactis* NZ9000 (B). (A) Starting from the outside: genes encoded on the top and bottom strand (first and fourth ring), tRNA and rRNA on the bottom and top strand (second and third ring). Genes are colored according to the corresponding functional categories shown on the right side. The fifth ring shows GC content deviations from the genomic average. The innermost ring shows GC skew; positive skew is shown in green, and negative skew is shown in purple.

**Additional file 5: Data S1B.** Genome sequencing and analysis of *L. lactis* 9k-5A. (B) Arrangement of 66 large scaffolds in *L. lactis* 9k-5A.

**Additional file 6: Data S2B.** Circular graph of *L. lactis* 9K-5A (A) and alignment with parent strain *L. lactis* NZ9000 (B). (B) Horizontal axis is the genome sequence of *L. lactis* NZ9000 and vertical axis is the genome sequence of *L. lactis* 9K-5A.

**Additional file 7: Figure S2.** Results of transcriptome analyses. (A–C) Volcano plot of different strains; green (downregulated) and red (upregulated) colors denote genes with significant changes in expression (DEGs), black color indicates no difference in gene expression. (D) Comparison of whole genome expression among all strains by RNA-Seq.

**Additional file 8: Table S2.** Results of transcriptome analysis.

**Additional file 9: Figure S3.** Heatmap profile and hierarchical cluster analysis of selected 93 genes expression in all the strains.

**Additional file 10: Table S3.** List of 93 DEGs in mutants.

**Additional file 11: Data S3A.** Results of significant enrichment of KEGG pathway in *L. lactis* 9k-5A. (A) Transcriptome analysis of 93 DEGs in mutants.

**Additional file 12: Data S3B.** Results of significant enrichment of KEGG pathway in *L. lactis* 9k-5A. Enrichment pathway map of genes involved in (B) glutamine metabolism, (C) and biosynthesis of valine and isoleucine. Map was downloaded from the KEGG server with our data mapping to the pathway (http://www.kegg.jp/kegg). Significant changes in expression are color-coded: red, up-regulated; green, down-regulated [43]. Glutamine, valine and isoleucine histidine are marked with red arrows.

**Additional file 13: Data S3C.** Results of significant enrichment of KEGG pathway in *L. lactis* 9k-5A. Enrichment pathway map of genes involved in (B) glutamine metabolism, (C) and biosynthesis of valine and isoleucine. Map was downloaded from the KEGG server with our data mapping to the pathway (http://www.kegg.jp/kegg). Significant changes in expression are color-coded: red, up-regulated; green, down-regulated [43]. Glutamine, valine and isoleucine histidine are marked with red arrows.

**Additional file 14: Figure S4.** RT-qPCR analysis of genes with higher expression level. *malD*: sugar ABC transporter permease; *purF*: phosphoribosylpyrophosphate amidotransferase; *galE*: UDP-glucose 4-epimerase; *galk*: galactokinase.

**Additional file 15: Table S4.** Comparison of the deletion efficiency of the two systems.

**Additional file 16: Figure S5.** Chromogenic reaction of (A) *L. lactis* MG1363 harboring pNZ5417Δ L4A on M17 medium containing chloramphenicol and gradient X-gal, (B) *L. lactis* MG1363 on M17 medium containing X-gal.

**Additional file 17: Table S5.** Primers utilized in this study.

## Data Availability

All data generated or analyzed during this study are included in this published article [and its additional files].

## Abbreviations

L-4A, L-5A: two large nonessential DNA regions
LB-CX: LB medium containing chloramphenicol and X-gal
GM17-CXN: GM17 medium containing chloramphenicol, X-gal, and nisin
DEGs: differentially expressed genes

## References

[1] Leroy F, De Vuyst L (2004). Lactic acid bacteria as functional starter cultures for the food fermentation industry. Trends Food Sci Technol.

[2] Morello E, Bermudez-Humaran L, Llull D, Sole V, Miraglio N, Langella P, Poquet I (2008). *Lactococcus lactis*, an efficient cell factory for recombinant protein production and secretion. J Mol Microbiol Biotechnol.

[3] Doerks T, Copley RR, Schultz J, Ponting CP, Bork P (2002). Systematic identification of novel protein domain families associated with nuclear functions. Genome Res.

[4] Hiroshi M, Hideo M, Tatsuro F (2011). *Escherichia coli* minimum genome factory. Biotechnol Appl Biochem.

[5] Mizoguchi H, Sawano Y, Kato J, Mori H (2008). Superpositioning of deletions promotes growth of *Escherichia coli* with a reduced genome. DNA Res.

[6] Hirokawa Y, Kawano H, Tanaka-Masuda K, Nakamura N, Nakagawa A, Ito M, Mori H, Oshima T, Ogasawara N (2013). Genetic manipulations restored the growth fitness of reduced-genome *Escherichia coli*. J Biosci Bioeng.

[7] GyöRgy P, Guy P, Tamás F, David F, Keil GM, Kinga U, Vitaliy K, Buffy S, Sharma SS, Monika DA (2006). Emergent properties of reduced-genome *Escherichia coli*. Science.

[8] Lieder S, Nikel PI, Lorenzo VD, Takors R (2015). Genome reduction boosts heterologous gene expression in *Pseudomonas putida*. Microb Cell Fact.

[9] Martínez-García E, Jatsenko T, Kivisaar M, de Lorenzo V (2015). Freeing *P. seudomonas putida* KT 2440 of its proviral load strengthens endurance to environmental stresses. Environ Microbiol.

[10] Morimoto T, Kadoya R, Endo K, Tohata M, Sawada K, Liu S, Ozawa T, Kodama T, Kakeshita H, Kageyama Y, Manabe K (2008). Enhanced recombinant protein productivity by genome reduction in *Bacillus subtilis*. DNA Res.

[11] Ara K, Ozaki K, Nakamura K, Yamane K, Sekiguchi J, Ogasawara N (2007). *Bacillus* minimum genome factory: effective utilization of microbial genome information. Biotechnol Appl Biochem.

[12] Hal A, Joel M, Elke N, Fink GR, Gregory S (2006). Engineering yeast transcription machinery for improved ethanol tolerance and production. Science.

[13] van Tilburg AY, Cao H, van der Meulen SB, Solopova A, Kuipers OP (2019). Metabolic engineering and synthetic biology employing *Lactococcus lactis* and *Bacillus subtilis* cell factories. Curr Opin Biotechnol.

[14] Zhu D, Fu Y, Liu F, Xu H, Saris PEJ, Qiao M (2017). Enhanced heterologous protein productivity by genome reduction in *Lactococcus lactis* NZ9000. Microb Cell Fact.

[15] Pinto JP, Zeyniyev A, Karsens H, Trip H, Lolkema JS, Kuipers OP, Kok J (2011). pSEUDO, a genetic integration standard for *Lactococcus lactis*. Appl Environ Microbiol.

[16] van Pijkeren J-P, Britton RA (2012). High efficiency recombineering in lactic acid bacteria. Nucleic Acids Res.

[17] Guo T, Xin Y, Zhang Y, Gu X, Kong J (2019). A rapid and versatile tool for genomic engineering in *Lactococcus lactis*. Microb Cell Fact.

[18] Berlec A, Škrlec K, Kocjan J, Olenic M, Štrukelj B (2018). Single plasmid systems for inducible dual protein expression and for CRISPR–Cas9/CRISPRi gene regulation in lactic acid bacterium *Lactococcus lactis*. Sci Rep.

[19] Zhu D, Zhao K, Xu H, Zhang X, Bai Y, Saris PE, Qiao M (2015). Construction of *thyA* deficient *Lactococcus lactis* using the Cre-*loxP* recombination system. Ann Microbiol.

[20] Lambert JM, Bongers RS, Kleerebezem M (2007). Cre-*lox*-based system for multiple gene deletions and selectable-marker removal in *Lactobacillus plantarum*. Appl Environ Microbiol.

[21] Koonin EV, Wolf YI (2008). Genomics of bacteria and archaea: the emerging dynamic view of the prokaryotic world. Nucleic Acids Res.

[22] Kurland CG, Canback B, Berg OG (2003). Horizontal gene transfer: a critical view. Proc Natl Acad Sci.

[23] Kurokawa M, Seno S, Matsuda H, Ying B-W (2016). Correlation between genome reduction and bacterial growth. DNA Res.

[24] Umenhoffer K, Draskovits GB, Nyerges AK, Karcagi I, Bogos BZ, Tímár E, Csörgő BL, Herczeg RB, Nagy IN, Fehér TS (2017). Genome-wide abolishment of mobile genetic elements using genome shuffling and CRISPR/Cas-assisted MAGE allows the efficient stabilization of a bacterial chassis. ACS Synth Biol.

[25] So Y, Park S-Y, Park E-H, Park S-H, Kim E-J, Pan J-G, Choi S-K (2017). A highly efficient CRISPR–Cas9-mediated large genomic deletion in *Bacillus subtilis*. Front Microbiol.

[26] Xin Y, Guo T, Mu Y, Kong J (2018). Coupling the recombineering to Cre-*lox* system enables simplified large-scale genome deletion in *Lactobacillus casei*. Microb Cell Fact.

[27] Xin Y, Guo T, Mu Y, Kong J (2018). A single-plasmid genome editing system for metabolic engineering of *Lactobacillus casei*. Front Microbiol.

[28] Wan X, Usvalampi AM, Saris PEJ, Takala TM (2016). A counterselection method for *Lactococcus lactis* genome editing based on class IIa bacteriocin sensitivity. Appl Microbiol Biotechnol.

[29] Christian S, Els D, Peter Ruhdal J, Jan M (2008). Plasmid pCS1966, a new selection/counterselection tool for lactic acid bacterium strain construction based on the *oroP* gene, encoding an orotate transporter from *Lactococcus lactis*. Appl Environ Microbiol.

[30] Li R, Takala TM, Qiao M, Xu H, Saris PEJ (2011). Nisin-selectable food-grade secretion vector for *Lactococcus lactis*. Biotechnol Lett.

[31] Tang X, Nakata Y, Li HO, Zhang M, Gao H, Fujita A, Sakatsume O, Ohta T, Yokoyama K (1994). The optimization of preparations of competent cells for transformation of *E. coli*. Nucleic Acids Res.

[32] Holo H, Nes IF (1989). High-frequency transformation, by electroporation, of *Lactococcus lactis* subsp. *cremoris* grown with glycine in osmotically stabilized media. Appl Environ Microbiol.

[33] Wan X, Li R, Saris PE, Takala TM (2013). Genetic characterisation and heterologous expression of leucocin C, a class IIa bacteriocin from *Leuconostoc carnosum* 4010. Appl Microbiol Biotechnol.

[34] Li R, Zhu HJ, Qian W, Fang X, Shi Z, Li Y, Li S, Shan G, Kristiansen K, Li S (2010). De novo assembly of human genomes with massively parallel short read sequencing. Genome Res.

[35] Delcher AL, Harmon D, Kasif S, White O, Salzberg SL (1999). Improved microbial gene identification with GLIMMER. Nucleic Acids Res.

[36] Lowe TM, Eddy SR (1997). tRNAscan-SE: a program for improved detection of transfer RNA genes in genomic sequence. Nucleic Acids Res.

[37] Yi-Sheng C, Chi-Huan C, Shwu-Fen P, Li-Ting W, Yu-Chung C, Hui-Chung W, Fujitoshi Y (2013). *Lactococcus taiwanensis* sp. nov., a lactic acid bacterium isolated from fresh cummingcordia. Int J Syst Evol Microbiol.

[38] Livak KJ, Schmittgen TD (2001). Analysis of relative gene expression data using real-time quantitative PCR and the 2^−ΔΔCT^ method. Methods.

[39] Woodcock DM, Crowther PJ, Doherty J, Jefferson S, Decruz E, Noyerweidner M, Smith SS, Michael MZ, Graham MW (1989). Quantitative evaluation of *Escherichia coli* host strains for tolerance to cytosine methylation in plasmid and phage recombinants. Nucleic Acids Res.

[40] Qiao M, Immonen T, Koponen O, Saris PEJ (1995). The cellular location and effect on nisin immunity of the NisI protein from *Lactococcus lactis* N8 expressed in *Escherichia coli* and *L. lactis*. FEMS Microbiol Lett.

[41] Gasson MJ (1983). Plasmid complements of *Streptococcus lactis* NCDO 712 and other lactic streptococci after protoplast-induced curing. J Bacteriol.

[42] Kuipers OP, de Ruyter PG, Kleerebezem M, de Vos WM (1998). Quorum sensing-controlled gene expression in lactic acid bacteria. J Biotechnol.

[43] Kanehisa M, Goto S (2000). KEGG: kyoto encyclopedia of genes and genomes. Nucleic acids Res.

