# Supplementary material for: Restructured Lactococcus lactis strains with emergent properties constructed by a novel highly efficient screening system
Source: Microb Cell Fact. 2019 Nov 14;18:198. doi: 10.1186/s12934-019-1249-z (PMC6854693; doi:10.1186/s12934-019-1249-z)
Supplement: Supplementary file 1 — Additional file 1: Table S1. Gene content of deleted DNA regions. [file 12934_2019_1249_MOESM1_ESM.docx]

**Table S1. Gene content of the deleted DNA regions**

| Item | Location | Note |
| --- | --- | --- |
| L4A DNA region (696671-706761) | | |
| LLNZ_03690 | 696671..697498 | /product="hypothetical protein" |
| LLNZ_03695 | 697488..697703 | /product="hypothetical protein" |
| LLNZ_03700 | 697733..698287 | /note="COG1961 Site-specific recombinases, DNA invertase Pin homologs" /product="DNA-invertase/resolvase" |
| LLNZ_03705 | Complement  (698631..699236) | /product="hypothetical protein" |
| LLNZ_03710 | Complement  (699249..699428) | /product="hypothetical protein" |
| LLNZ_03715 | Complement  (699525..700379 | /note="COG2801 Transposase and inactivated derivatives" /product="transposase for insertion sequence element IS981F" |
| LLNZ_03720 | Complement  (700376..700543) | /note="COG2963 Transposase and inactivated derivatives" /product="IS981 transposase A" |
| LLNZ_03725 | 700608..701249 | /product="hypothetical protein" |
| LLNZ_03730 | 701492..701755 | /product="hypothetical protein" |
| LLNZ_03735 | Complement  (701860..702540) | /note="COG3316 Transposase and inactivated derivatives" /product="transposase for insertion sequence element IS946" |
| LLNZ_03740 | Complement  (702572..703441) | /note="COG4584 Transposase and inactivated derivatives" /product="transposase for insertion sequence element IS712H" |
| LLNZ_03745 | 703807..704460 | /note="COG0476 Dinucleotide-utilizing enzymes involved in molybdopterin and thiamine biosynthesis family 2" /product="putative nucleotide binding protein" |
| LLNZ_03750 | 704453..705613 | /note="COG4166 ABC-type oligopeptide transport system, periplasmic component" /product="ABC transporter substrate-binding protein" |
| LLNZ_03755 | 705650..705910 | /note="COG2963 Transposase and inactivated derivatives" /product="IS981 transposase A" |
| LLNZ_03760 | 705907..706761 | /note="COG2801 Transposase and inactivated derivatives" /product="transposase" |
|  |  |  |
| L5A DNA region (2073682-2095306) | | |
| LLNZ_10745 | Complement  (2073682..2078829) | /note="COG5283 Phage-related tail protein" |
| LLNZ_10750 | Complement  (2079052..2079468) | /product="phage tail component" |
| LLNZ_10755 | Complement  (2079613..2080206) | /product="phage major tail protein" |
| LLNZ_10760 | Complement  (2080237..2080632) | /product="hypothetical protein" |
| LLNZ_10765 | Complement  (2080629..2081135) | /product="hypothetical protein" |
| LLNZ_10770 | Complement  (2081137..2081487) | /product="Phage protein, head-tail joining protein" |
| LLNZ_10775 | Complement  (2081462..2081785) | /product="hypothetical protein" |
| LLNZ_10780 | Complement  (2081986..2083200) | /product="putative major head protein precursor" |
| LLNZ_10785 | Complement  (2083212..2083916) | /note="COG0740 Protease subunit of ATP-dependent Clp proteases" /product="ATP dependent Clp protease" |
| LLNZ_10790 | Complement  (2083962..2085140) | /note="COG4695 Phage-related protein" /product="prophage pi3 protein 24" |
| LLNZ_10795 | Complement  (2085137..2085328) | /product="putative head-tail joining protein" |
| LLNZ_10800 | Complement  (2085315..2086913) | /note="COG4626 Phage terminase-like protein, large subunit" /product="putative terminase large subunit" |
| LLNZ_10815 | Complement  (2088262..2088546) | /note="COG4626 Phage terminase-like protein, large subunit" /product="terminase large subunit" |
| LLNZ_10820 | Complement  (2088536..2088934) | /note="COG3747 Phage terminase, small subunit" /product="phage terminase small subunit" |
| LLNZ_10825 | Complement  (2089116..2089634) | /note="COG1403 Restriction endonuclease" /product="hypothetical protein" |
| LLNZ_10830 | Complement  (2089638..2089928) | /product="hypothetical protein" |
| LLNZ_10835 | Complement  (2090305..2090706) | /product="hypothetical protein" |
| LLNZ_10840 | Complement  (2090789..2090974) | /product="hypothetical protein" |
| LLNZ_10845 | Complement  (2090971..2091279) | /product="hypothetical protein" |
| LLNZ_10850 | Complement  (2091281..2091460) | /product="hypothetical protein" |
| LLNZ_10855 | Complement  (2091462..2091635) | /product="hypothetical protein" |
| LLNZ_10860 | Complement  (2091711..2092469) | /note="COG1484 DNA replication protein" /product="putative transposase helper protein for IS712H" |
| LLNZ_10865 | Complement  (2092481..2093704) | /note="COG4584 Transposase and inactivated derivatives" /product="transposase for insertion sequence element IS712H" |
| LLNZ_10870 | Complement  (2093760..2093903) | /product="hypothetical protein" |
| LLNZ_10875 | 2094264..2094473 | /product="hypothetical protein" |
| LLNZ_10880 | Complement  (2094522..2094722) | /product="hypothetical protein" |
| LLNZ_10885 | Complement  (2094719..2094949) | /product="hypothetical protein" |
| LLNZ_10890 | Complement  (2094968..2095306) | /product="hypothetical protein" |
