# Supplementary figures and images for: Restructured Lactococcus lactis strains with emergent properties constructed by a novel highly efficient screening system
Source: Microb Cell Fact. 2019 Nov 14;18:198. doi: 10.1186/s12934-019-1249-z (PMC6854693; doi:10.1186/s12934-019-1249-z)

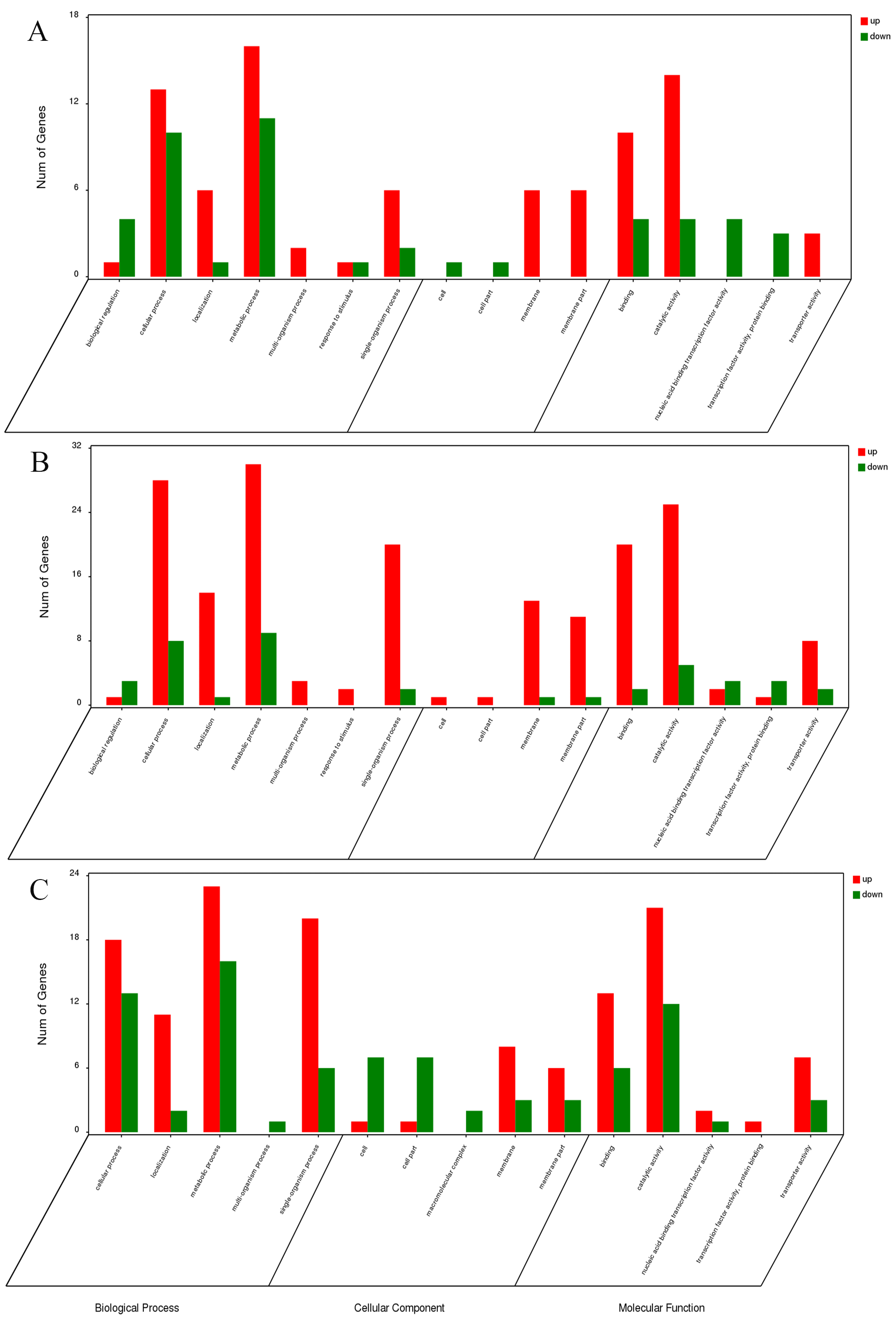

Supplement: Supplementary file 2 — Additional file 2: Figure S1. Go terms analysis for all the DEGs of all mutants. (A) Go terms of L. lactis NZ9000-VS-L. lactis 9k-4A, (B) L. lactis NZ9000-VS-L. lactis 9k-5A, (C) L. lactis 9k-4A-VS-L. lactis 9k-5A. [file 12934_2019_1249_MOESM2_ESM.tif]

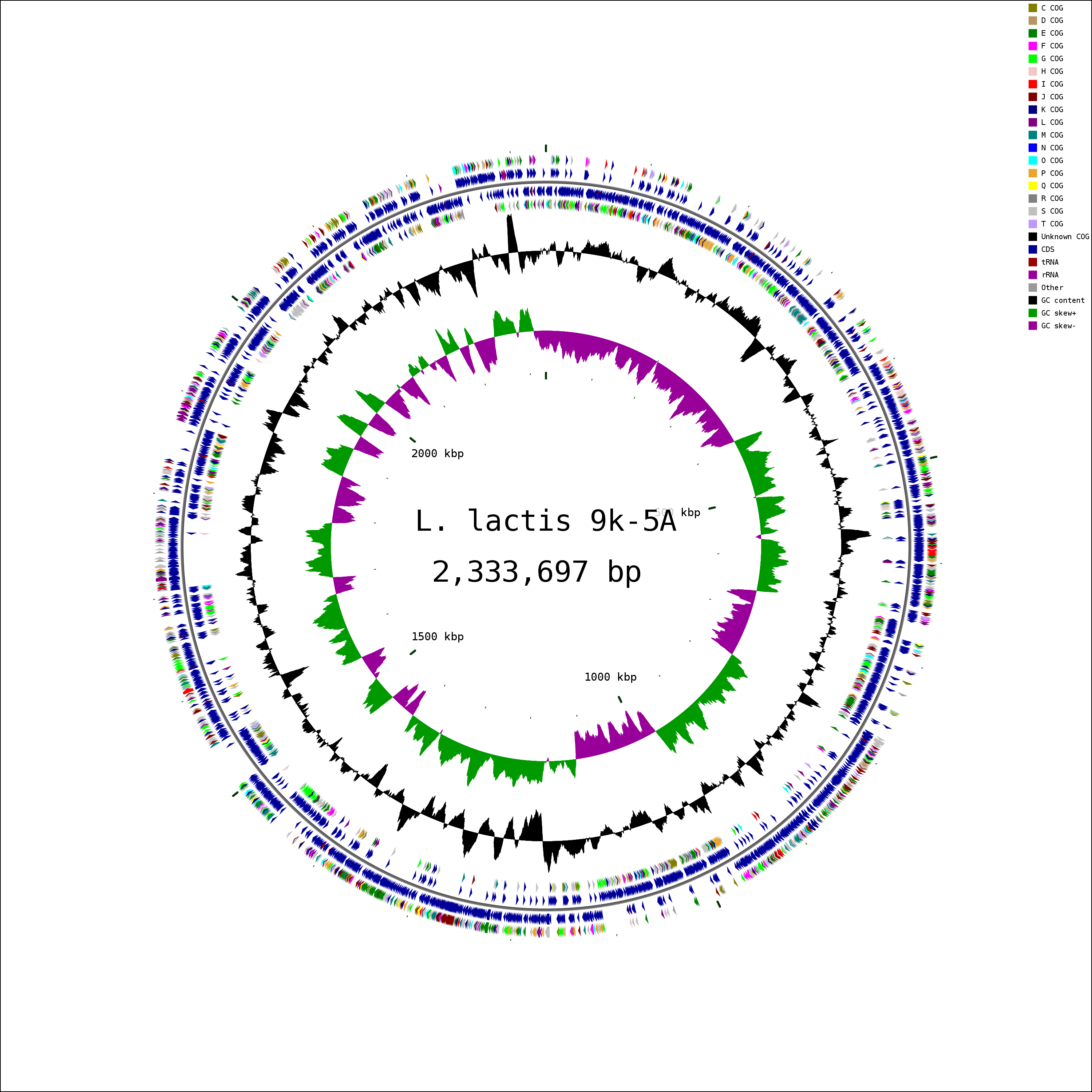

Supplement: Supplementary file 4 — Additional file 4: Data S2A. Circular graph of L. lactis 9K-5A (A) and alignment with parent strain L. lactis NZ9000 (B). (A) Starting from the outside: genes encoded on the top and bottom strand (first and fourth ring), tRNA and rRNA on the bottom and top strand (second and third ring). Genes are colored according to the corresponding functional categories shown on the right side. The fifth ring shows GC content deviations from the genomic average. The innermost ring shows GC skew; positive skew is shown in green, and negative skew is shown in purple. [file 12934_2019_1249_MOESM4_ESM.png]

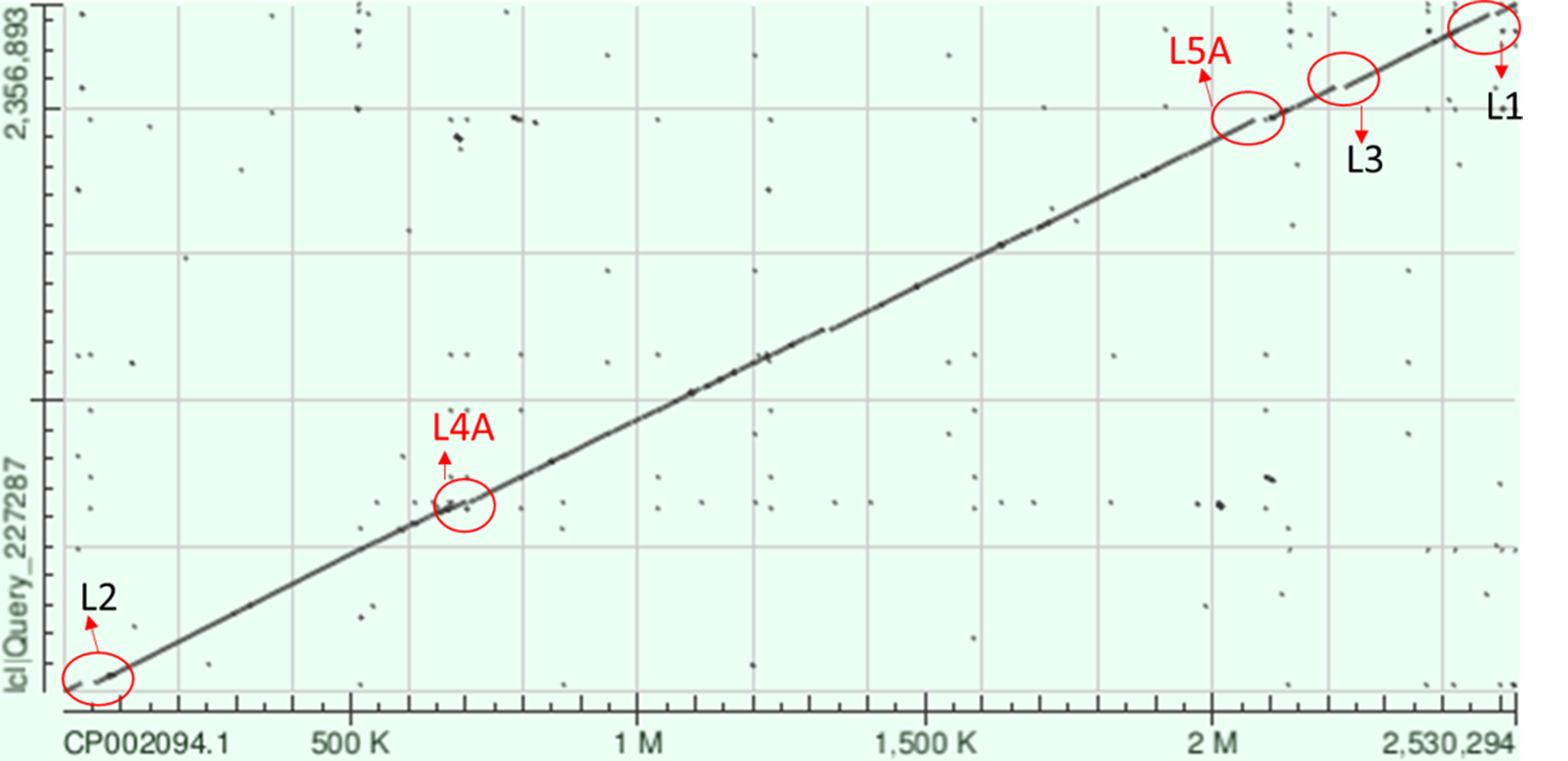

Supplement: Supplementary file 6 — Additional file 6: Data S2B. Circular graph of L. lactis 9K-5A (A) and alignment with parent strain L. lactis NZ9000 (B). (B) Horizontal axis is the genome sequence of L. lactis NZ9000 and vertical axis is the genome sequence of L. lactis 9K-5A. [file 12934_2019_1249_MOESM6_ESM.png]

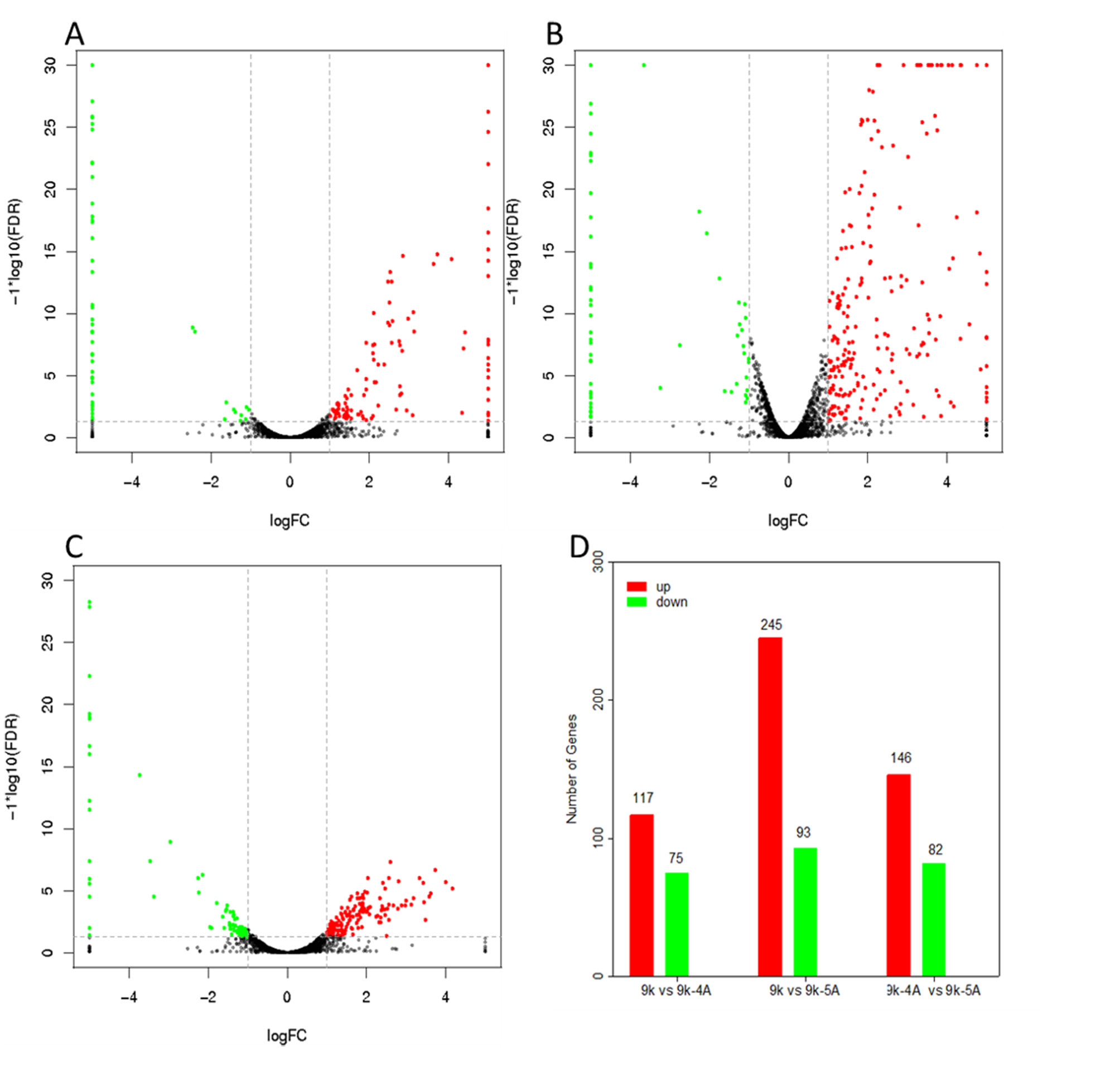

Supplement: Supplementary file 7 — Additional file 7: Figure S2. Results of transcriptome analyses. (A–C) Volcano plot of different strains; green (downregulated) and red (upregulated) colors denote genes with significant changes in expression (DEGs), black color indicates no difference in gene expression. (D) Comparison of whole genome expression among all strains by RNA-Seq. [file 12934_2019_1249_MOESM7_ESM.tif]

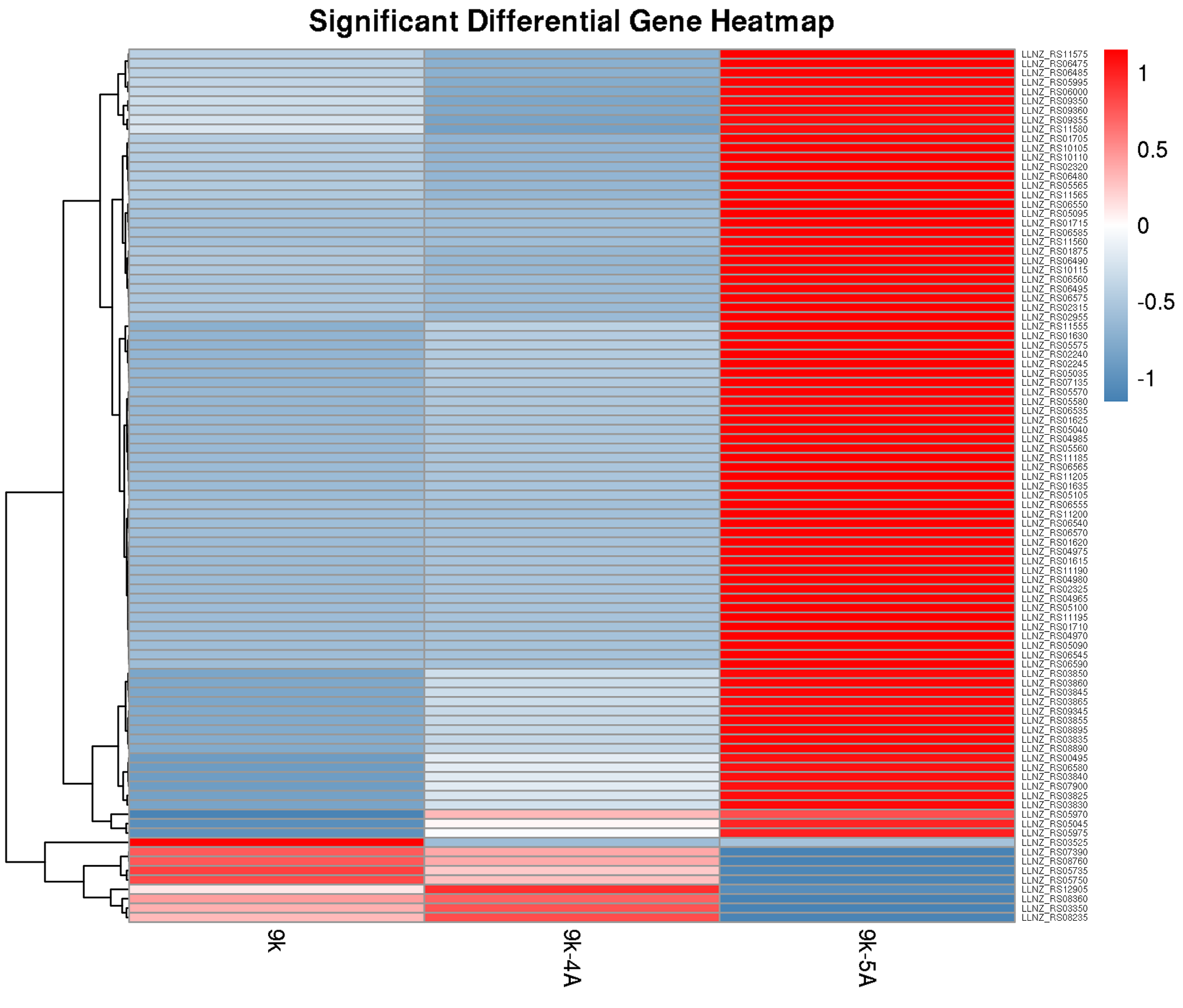

Supplement: Supplementary file 9 — Additional file 9: Figure S3. Heatmap profile and hierarchical cluster analysis of selected 93 genes expression in all the strains. [file 12934_2019_1249_MOESM9_ESM.tif]

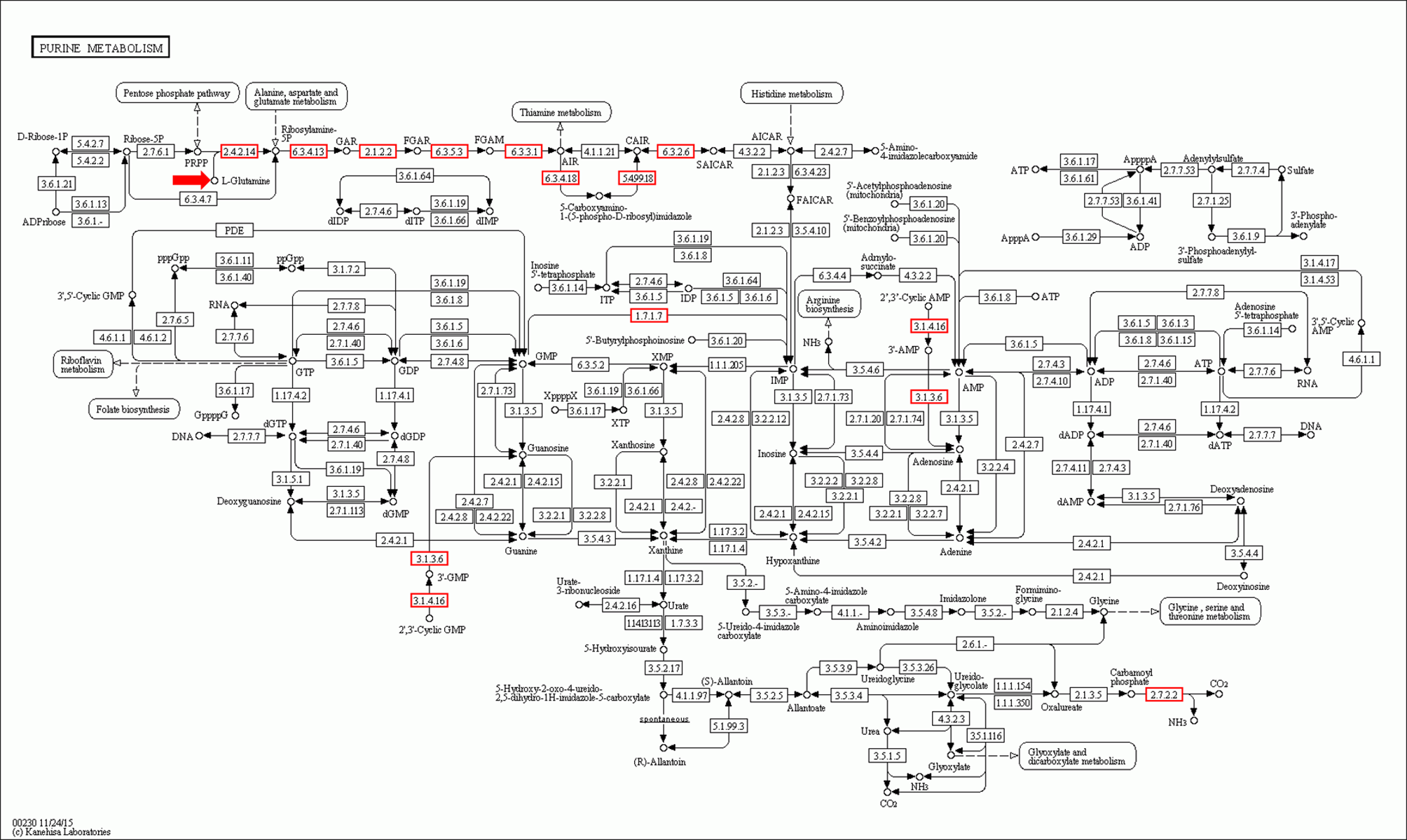

Supplement: Supplementary file 12 — Additional file 12: Data S3B. Results of significant enrichment of KEGG pathway in L. lactis 9k-5A. Enrichment pathway map of genes involved in (B) glutamine metabolism, (C) and biosynthesis of valine and isoleucine. Map was downloaded from the KEGG server with our data mapping to the pathway (http://www.kegg.jp/kegg). Significant changes in expression are color-coded: red, up-regulated; green, down-regulated [43]. Glutamine, valine and isoleucine histidine are marked with red arrows. [file 12934_2019_1249_MOESM12_ESM.tif]

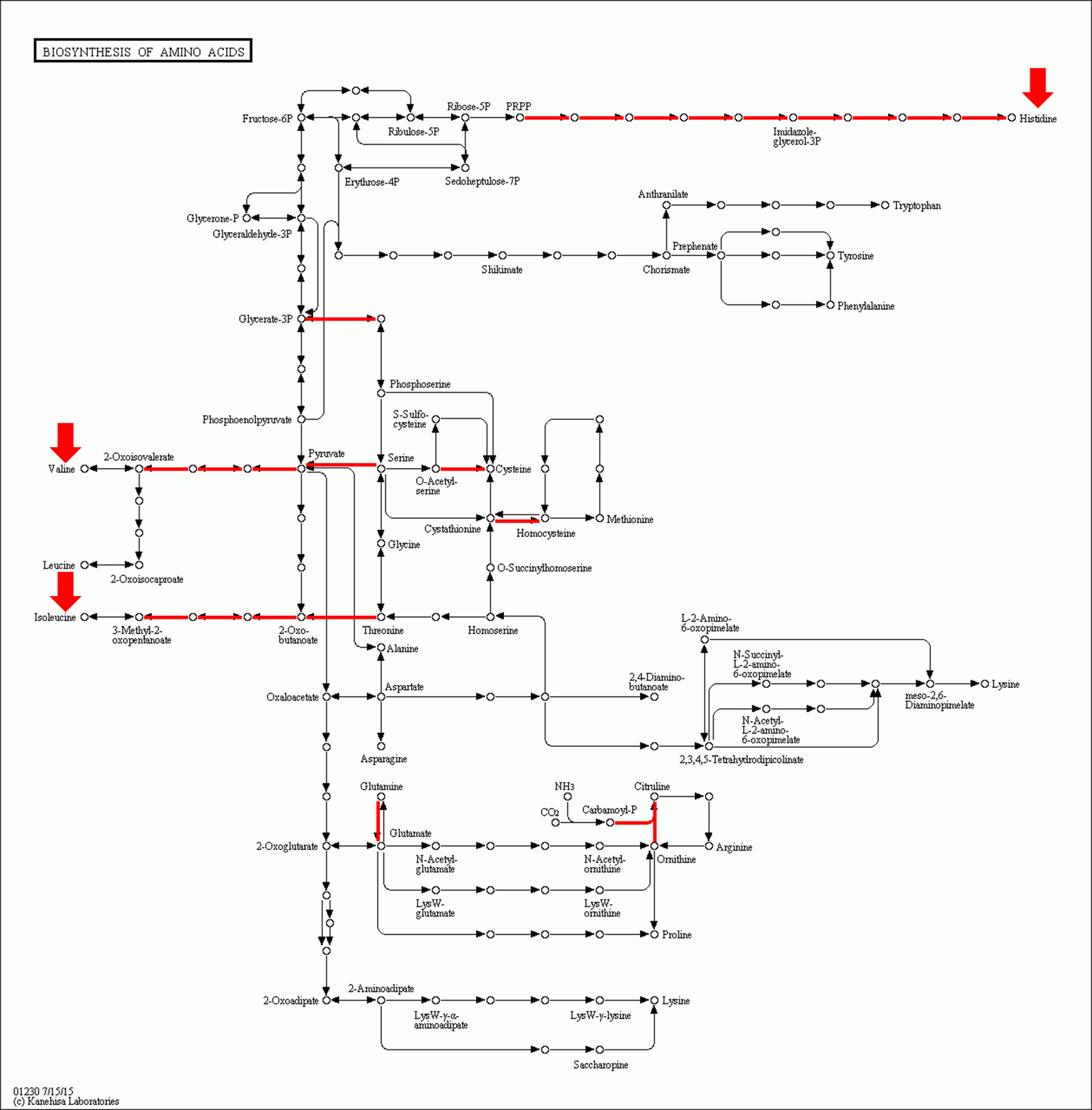

Supplement: Supplementary file 13 — Additional file 13: Data S3C. Results of significant enrichment of KEGG pathway in L. lactis 9k-5A. Enrichment pathway map of genes involved in (B) glutamine metabolism, (C) and biosynthesis of valine and isoleucine. Map was downloaded from the KEGG server with our data mapping to the pathway (http://www.kegg.jp/kegg). Significant changes in expression are color-coded: red, up-regulated; green, down-regulated [43]. Glutamine, valine and isoleucine histidine are marked with red arrows. [file 12934_2019_1249_MOESM13_ESM.tif]

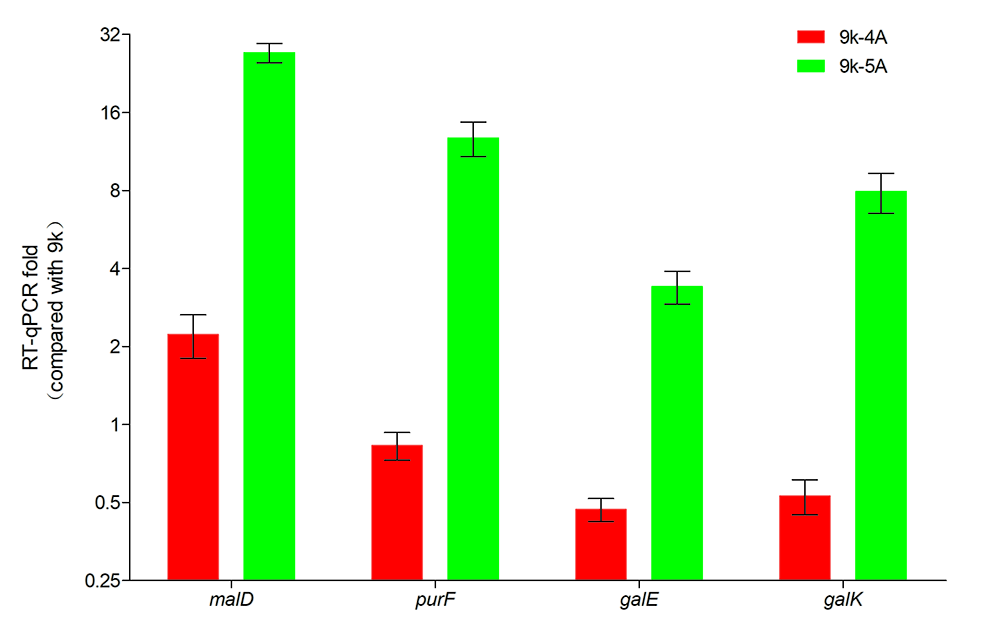

Supplement: Supplementary file 14 — Additional file 14: Figure S4. RT-qPCR analysis of genes with higher expression level. malD: sugar ABC transporter permease; purF: phosphoribosylpyrophosphate amidotransferase; galE: UDP-glucose 4-epimerase; galk: galactokinase. [file 12934_2019_1249_MOESM14_ESM.tif]

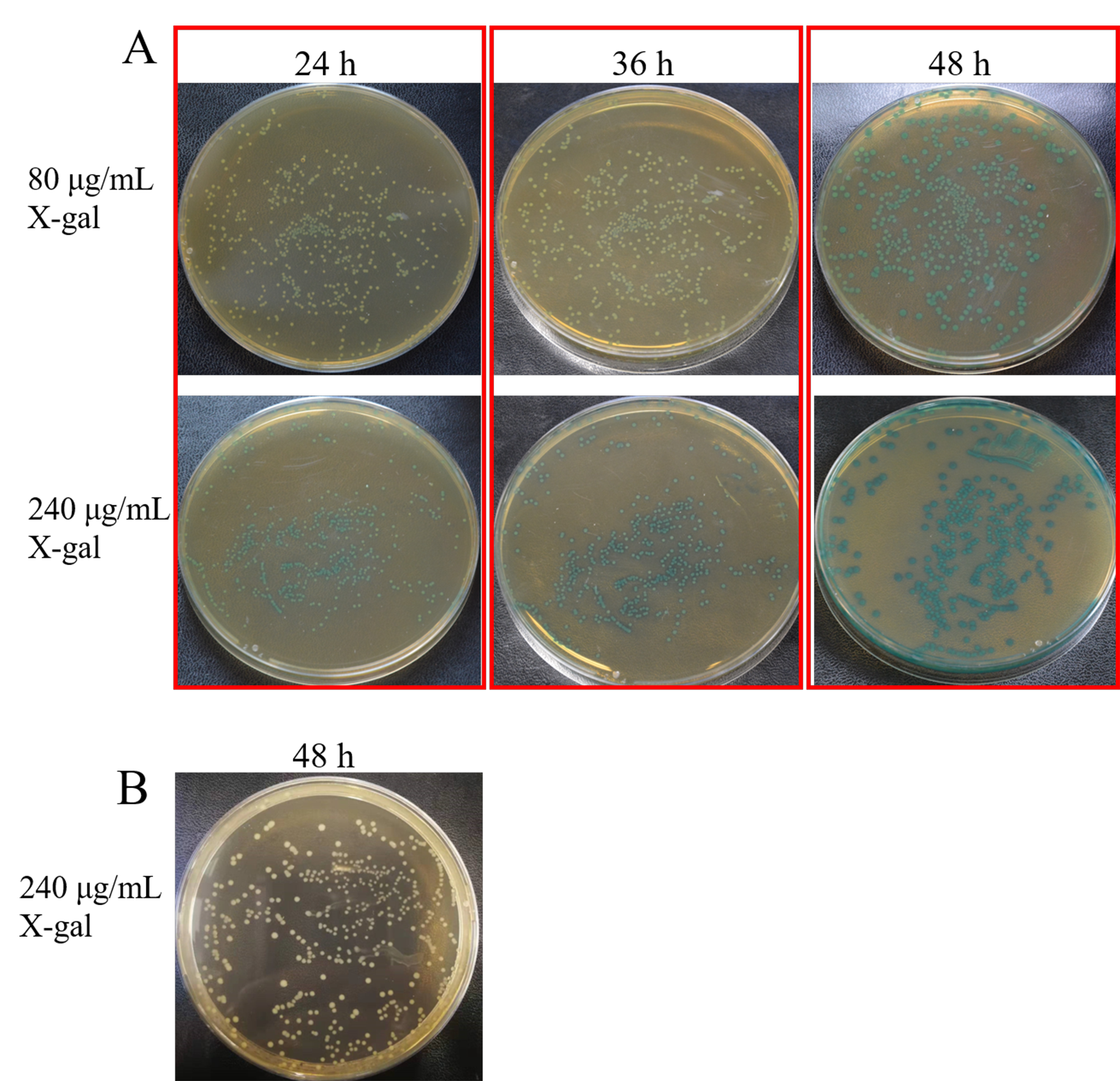

Supplement: Supplementary file 16 — Additional file 16: Figure S5. Chromogenic reaction of (A) L. lactis MG1363 harboring pNZ5417Δ L4A on M17 medium containing chloramphenicol and gradient X-gal, (B) L. lactis MG1363 on M17 medium containing X-gal. [file 12934_2019_1249_MOESM16_ESM.tif]
