# Supplementary material for: Restructured Lactococcus lactis strains with emergent properties constructed by a novel highly efficient screening system
Source: Microb Cell Fact. 2019 Nov 14;18:198. doi: 10.1186/s12934-019-1249-z (PMC6854693; doi:10.1186/s12934-019-1249-z)
Supplement: Supplementary file 15 — Additional file 15: Table S4. Comparison of the deletion efficiency of the two systems. [file 12934_2019_1249_MOESM15_ESM.docx]

**Table S4.** **Comparison of the deletion efficiency of the two systems**

| **Steps** | **Times(day)** | |
| --- | --- | --- |
|  | **pNZ5319/pNZTS-Cre gene deletion system** | **single-vector gene deletion system** |
| (i) Construct deletion vector and select first-crossover recombination | 4 | 4 |
| (ii) Culture and screen second-crossover recombination | 20 | 7 |
| (iii) Deletion of the chloromycetin resistance marker and elimination of temperature-sensitive plasmid pNZTS-Cre | 10 | 10 |
