# Supplementary material for: Restructured Lactococcus lactis strains with emergent properties constructed by a novel highly efficient screening system
Source: Microb Cell Fact. 2019 Nov 14;18:198. doi: 10.1186/s12934-019-1249-z (PMC6854693; doi:10.1186/s12934-019-1249-z)
Supplement: Supplementary file 17 — Additional file 17: Table S5. Primers utilized in this study. [file 12934_2019_1249_MOESM17_ESM.docx]

**Table S5. Primers utilized in this study**

| Primer | Sequence (5´ to 3´) | Restriction sites |
| --- | --- | --- |
| For construction of the plasmid pNZ5525 | | |
| P_nisZ_-F | ga **AGATCT** AGTCTTATAACTATACTGACAATAGAAAC | *Bgl*II |
| P_nisZ_-R | ATCAATCATTGTCATCATAGCCATTGAGTGCCTCCTTATAATTTAT |  |
| LacZ-F | ATAAATTATAAGGAGGCACTCAATGGCTATGATGACAATGATTGAT |  |
| LacZ-R | acat **GCATGC**TTTGGATCATCACGTTAATTTAAAAC | *Sph*I |
| For construction of the large-scale gene KO vector | | |
| L4A UP-F | ccg **GTTTAAAC** GAGTTTCCCCAAGGTTTC | *Pme*I |
| L4A UP-R | ccc **ATTTAAAT** TCGGCAGATTCTCCATTA | *Swa*I |
| L4A DP-F | ccc **CCCGGG** GCCAGTCTTGAATCTTGG | *Sma*I |
| L4A DP-R | ga **AGATCT** CTTGAACGTGCCCTTTAT | *Bgl*II |
| L5A UP-F | ccg **GTTTAAAC** GATTTGGTACTTTCCCGTCA | *Pme*I |
| L5A UP-R | ccc **ATTTAAAT**CCGCAACTATTAATAAGACAC | *Swa*I |
| L5A DP-F | ccc **CCCGGG** TATTTGGTAGTCTTGCACGAT | *Sma*I |
| L5A DP-R | ga **AGATCT** ACTAAGCGAATTTGAATCAGT | *Bgl*II |
| For identification of the large-scale gene deletion strain | | |
| Int4 A-F | AAAATCGAGGTTCTGTTG |  |
| Int4 A-R | ATACGGGAGTCATCTTTG |  |
| Out4 A-F | AGTAGAAAAGCACATCAA |  |
| Out4 A-R | TGAACCTAAAGCTCATTG |  |
| Int-5 A-F | CGCCAGAAGTTGGTAGTG |  |
| Int-5 A-R | ACCCCATCTTGTAGCAGT |  |
| Out5 A-F | CCGCCATAAACATATACCTT |  |
| Out5 A-R | AGATGTAGCACAGATACAAC |  |
| For the RT-qPCR | | |
| Q-rpoB-F | ACACCGTACAAGACGCAGTT |  |
| Q-rpoB-R | CCCGCAAAGTTGTCGATTGG |  |
| Q-malD-F | TAGGTGGAGCTGGCTTAGGT |  |
| Q-malD-R | CGTGAAAGTGGCAGGGTGAT |  |
| Q-purF-F | CTACGCTGAAGAATCGGGCT |  |
| Q-purF-R | TTCATGCGAACGCCTTGTTC |  |
| Q-galE-F | CGTGGTTACGATGTGGCAGT |  |
| Q-galE-R | AGCATGGTCCCGTACATCTC |  |
| Q-galK-F | TGTGCAATCGCTCTTGTTGC |  |
| Q-galK-R | CTAGCTTTGTTGAGCCAGAACC |  |
